# Changes in multimorbidity burden over a 3–5 year period among people with HIV

**DOI:** 10.3389/fsysb.2023.1136999

**Published:** 2023-02-27

**Authors:** Luxsena Sukumaran, Davide De Francesco, Alan Winston, Patrick W. G. Mallon, Nicki Doyle, Jane Anderson, Marta Boffito, Ian Williams, Frank A. Post, Jaime Vera, Memory Sachikonye, Margaret A. Johnson, Caroline A. Sabin

**Affiliations:** ^1^ Institute for Global Health, University College London, London, United Kingdom; ^2^ National Institute for Health and Care Research (NIHR) Health Protection Research Unit (HPRU) in Blood Borne and Sexually Transmitted Infections at University College London, London, United Kingdom; ^3^ Department of Anesthesiology Perioperative and Pain Medicine, Stanford University, Stanford, CA, United States; ^4^ Imperial College London, London, United Kingdom; ^5^ University College Dublin, Dublin, Ireland; ^6^ Homerton University Hospital, London, United Kingdom; ^7^ Chelsea and Westminster Healthcare NHS Foundation Trust, London, United Kingdom; ^8^ King’s College Hospital NHS Foundation Trust, London, United Kingdom; ^9^ Brighton and Sussex Medical School, Brighton, United Kingdom; ^10^ UK Community Advisory Board (UK-CAB), London, United Kingdom; ^11^ Ian Charleson Day Centre, Royal Free NHS Trust, London, United Kingdom

**Keywords:** HIV, human immunodeficiency virus, multimorbidity (MM), comorbidity [MeSH], multimorbidity patterns, longitudinal, principal component analysis, PCA

## Abstract

**Introduction:** As people living with HIV age, the increasing burden of multimorbidity poses a significant health challenge. The aims of this study were to identify common patterns of multimorbidity and examine changes in their burden, as well as their associations with risk factors, over a 3–5 year period in people with HIV, enrolled in the Pharmacokinetic and clinical Observations in PeoPle over fiftY (POPPY) study.

**Methods:** Common multimorbidity patterns were identified in POPPY participants with HIV using principal component analysis, based on Somers’ D statistic. Multimorbidity burden scores were calculated for each participant/pattern at study entry/follow-up and were standardised relative to the mean in the sample at baseline (scores >0 thus reflect a greater number of comorbidities relative to the mean). Two multivariable linear regression models were fitted to examine the associations between risk factors and burden z-scores at baseline and change in z-scores over a 3–5 year period.

**Results:** Five patterns were identified among the 1073 POPPY participants with HIV {median age [interquartile range (IQR)], 52 (47–59) years; 85% male and 84% white}: Cardiovascular diseases (CVDs), Sexually transmitted diseases (STDs), Neurometabolic, Cancer and Mental-gastro-joint. The multivariable linear regression showed that older age, behavioural factors (i.e., body mass index (BMI), history of injection drug use, current recreational drug use and sex between men), and HIV-specific factors (i.e., duration since HIV diagnosis and a prior AIDS diagnosis) were associated with higher multimorbidity burden at baseline. However, only three of the factors (age, BMI and duration since HIV diagnosis) were significantly associated with an increase in burden across specific patterns over time.

**Discussion:** Key modifiable and non-modifiable factors contributing to an increase in burden of multimorbidity were identified. Our findings may inform the development of more targeted interventions and guidelines to effectively prevent and manage the rising burden of multimorbidity in people with HIV.

## Introduction

The widespread use of antiretroviral therapy (ART) has dramatically reduced the risk of AIDS-related morbidity and mortality and improved the overall survival of people with HIV (May et al., 2014). As a result, those living with HIV are experiencing a similar life expectancy to that of HIV-negative individuals. Currently, around one in three individuals accessing HIV care in the UK are aged ≥ 50 years (Kirwan et al., 2016), a proportion that is projected to increase to 54% by 2028 (Yin et al., 2015). As people with HIV are growing older, multimorbidity, defined as the co-existence of two or more health conditions (Boyd and Fortin, 2010), is becoming increasingly prevalent. Several studies have reported that people with HIV have a greater risk of developing comorbidities traditionally associated with aging, including cardiovascular, metabolic, bone and neurodegenerative disorders, compared with their HIV-negative counterparts (Guaraldi et al., 2011; Althoff et al., 2015). Multimorbidity presents a significant health challenge with far-reaching implications for people with HIV, particularly as current guidelines are often tailored to address the management of individual comorbidities (EACS, 2022; Waters et al., 2022) and rarely address the complex needs of individuals with multiple morbidities and their associated impacts, which include polypharmacy, drug-drug interactions and adverse therapeutic effects. Consequently, people with HIV may be at a higher risk of adverse health outcomes associated with multimorbidity, such as poorer quality of life and higher healthcare costs/utilisation (Gebo, 2008; Nachega et al., 2012; Rodriguez-Penney et al., 2013; Van Duin et al., 2017).

Certain health conditions are more likely to co-occur among people with HIV due to shared causes and risk factors, with likely contributions from HIV-mediated inflammatory processes (e.g., chronic inflammation and systemic immune activation) and long-term exposure to ART (Deeks and Phillips, 2009; Althoff et al., 2015; Guaraldi et al., 2017; Wong et al., 2018). Behavioural and/or environmental factors may also play an important role. For example, a higher prevalence of smoking is reported in people with HIV compared to the general population, a behaviour that is recognised as a risk factor for multiple conditions including lung cancer and obstructive lung disorders (Rossouw et al., 2015). Additionally, chronic inflammation has been linked to the development of both cardiovascular disease and neurocognitive decline (Deeks et al., 2013; Babu et al., 2019).

Over the last decade, the clustering of comorbidities has been increasingly explored using data-driven approaches which, compared to other measures (e.g., simple counts and weighted indices), can identify non-random co-occurrence of conditions (De Francesco et al., 2020) and improve our understanding of common clusters (patterns), associations and interactions between comorbidities. This, in turn, will allow us to identify patterns that pose the greatest burden where targeted prevention and treatment interventions may be most appropriate. To our knowledge, only four studies have investigated multimorbidity patterns in people with HIV using a data-driven approach. A recent review highlighted that certain patterns among HIV populations were also generally reported in the general population, such as those including CVDs, metabolic disorders and mental health problems (De Francesco et al., 2020). In addition, novel patterns, including STDs and conditions related to substance use (alcohol, recreational drugs, tobacco) or their complications, were also identified. We previously identified six common patterns among people with HIV receiving clinical care in the UK and Ireland (enrolled in the Pharmacokinetic and clinical Observations in PeoPle over fiftY (POPPY) study between 2013 and 2016) (De Francesco et al., 2018). However, the list of comorbidities (65 in total) was selected using a prevalence threshold of ≥ 1.5% in the study population. Use of this threshold may have failed to accurately capture key morbidities in this population, such as rarer and/or complex conditions that pose a greater multimorbidity burden. Furthermore, how the burden of specific patterns evolves over time remains unclear. Here we aim to: 1) re-identify multimorbidity patterns in POPPY participants with HIV, using an updated wider list of comorbidities; 2) examine changes in the burden of multimorbidity patterns over a 3–5-year follow up period; and 3) investigate the relative contributions of socio-demographic, lifestyle and clinical factors to these changes in each pattern over time.

## Methods

### Study design, setting and population

The POPPY study is a prospective cohort study that includes three groups: “older” (aged ≥50 years) and “younger” (18–49 years) groups of people with HIV (the latter being frequency-matched to the older group on gender, ethnicity, sexual orientation, and participating clinic), and a group of HIV-negative controls aged ≥ 50 years. The present analyses were restricted to participants with HIV as a control group was not directly relevant to our aims here. Participants with HIV (*n* = 1,073) were recruited from 8 outpatient clinics (7 in England and 1 in Ireland) between April 2013 and January 2016 (Bagkeris et al., 2018). Briefly, participants eligible for inclusion were selected using the following criteria to reflect the UK HIV population in clinical care: 1) documented presence of HIV infection; 2) white or black-African ethnicity; 3) likely route of HIV acquisition *via* sexual exposure; and 4) the ability to comprehend the study information leaflet. All study participants provided written informed consent. The study was approved by the UK National Research Ethics Service (NRES; Fulham, London, United Kingdom, number 12/LO/1409).

### Data on comorbidities

At baseline (April 2013–January 2016) and follow-up (May 2015–February 2018), information on the presence/absence of comorbidities was collected using self-reported medical history supplemented using information on concomitant medication (non-antiretroviral) and healthcare utilisation data (including visits to general practitioners, hospitals, psychiatrists/psychologists, specialists, and use of ambulance/hospital transport). Participants were asked to report whether they had ever developed any of 52 health conditions (from 17 pathophysiological systems) from a detailed list and to add any other conditions experienced (using free-text) that were not included in the list. Conditions that were reported by < 1.0% of participants were excluded, giving a total of 73 comorbidities for the present analysis (Supplementary Table S1).

### Risk factors associated with changes in multimorbidity burden

A range of covariates (demographic, lifestyle and clinical) recorded at baseline were included based on previous literature on the determinants of multimorbidity identified among cohorts of people with HIV: Age (per 10-year increment), sex (male vs. female), race (black African vs. white ethnicity), sexual orientation (men who have sex with men (MSM) or heterosexual), body mass index (BMI, per 1-kg/m^2^ increment), current smoking (current smoker, ex- and non-smoker) and alcohol consumption (current, ex- and no-use), current recreational drug use (yes or no within the 6 months preceding study visit) and history of injection drug use (IDU, yes or no). The following HIV-specific factors, which were obtained *via* linkage with the UK CHIC study [16] and the Mater Misericordiae University Hospital Infectious Diseases cohort for participants recruited in Ireland, were also included: nadir CD4^+^ T-cell count (per 100 cells/μL increment), years since HIV diagnosis (per 5-year increment) and any prior AIDS-related event (yes or no). Further information on the statistical properties of the included variables can be found in our previous publication (De Francesco et al., 2018).

### Statistical analysis

#### Descriptive analysis

Baseline demographic, clinical and lifestyle characteristics of included participants were described. Categorical data are presented as frequencies and proportions (%) and continuous data as median and interquartile range (IQR).

#### Patterns of multimorbidity

As previously described (De Francesco et al., 2018), common multimorbidity patterns were identified among all POPPY participants with HIV (*n* = 1,073) using principal component analysis (PCA) (Jolliffe, 2014). Briefly, Somers’ D statistic was used to assess pairwise associations between the 73 comorbidities at baseline, as current evidence suggests that this approach is more accurate in detecting non-random associations between comorbidities compared to other agreement measures (Ng, 2015; De Francesco et al., 2018). PCA was then applied to the matrix with the pairwise associations to reduce the original set of variables to a smaller set of principal components (PCs), whilst retaining as much of the variability in the dataset as possible. These PCs can be interpreted as patterns of multimorbidity, i.e., non-random groups of comorbidities. In order to allow multiple patterns to be present within one individual, an *oblimin* rotation was used in the PCA. To be consistent with our previous publication, a correlation ≥ 0.40 was used to determine comorbidities that were significantly associated with a pattern and were subsequently used to determine the label of that pattern.

#### Burden of multimorbidity patterns over time

The multimorbidity patterns were assessed over time by calculating burden scores for each participant and each pattern at baseline and follow-up. The analysis excluded those who were lost to follow-up (*n* = 238) or who had missing clinical data (BMI (*n* = 7); nadir CD4^+^ T-cell count (*n* = 36); years since HIV diagnosis (*n* = 4)). Descriptive characteristics were compared between those who were included (*n* = 788) and those excluded (*n* = 285) from analyses (Supplementary Table S2) with no large differences in the characteristics of the two groups.

Multimorbidity burden scores were determined using data on the comorbidities (presence/absence) and the positive coefficients (loadings) returned by the PCA (Supplementary Figures S1–S4) on baseline data. The scores were standardised relative to the mean in the sample at baseline, with negative z-scores denoting scores that were lower than the average from the baseline population. Scores are proportional to the number of comorbidities included within a pattern, with higher scores representing a greater number of comorbidities in an individual. All comorbidities (except *STDs*) were assumed to be chronic in nature and therefore were assessed cumulatively, i.e., any comorbidities reported by individuals at baseline were carried forward to follow-up. Changes in the burden of the ‘*STD*’ pattern over follow-up was not considered since this includes comorbidities of acute nature and were not of clinical interest/significance for the present analyses. Negative PCA loadings were excluded as they were relatively small in magnitude and were judged by authors to lack clinical relevance when compared with the comorbidities that were significantly associated with the pattern (i.e., ≥0.4 loading); this ensured that follow-up scores could not decrease due to the new onset of a condition with a negative weighting. We did not re-run the PCA at follow-up as our aim was to investigate changes in the burden scores over time and thus needed comparability of the scores at the two timepoints.

Two multivariable linear regression models were fitted to examine the associations between the previously described risk factors and burden z-scores at baseline and change in z-scores over the 3–5 year period. An additional analysis was also conducted in a subset of the included participants who had available data on CD4/CD8 ratio (*n* = 770), to explore its association with baseline and changes in burden z-scores. All associations are reported as coefficients with associated 95% confidence intervals (CI). All analyses were conducted using R (version 4.2.4). A significance level of *p* < .05 guided statistical interpretation.

## Results

### Characteristics of study participants

The socio-demographic and HIV-related characteristics of the 788 participants with complete data are summarised in Table 1. Participants were predominantly male (85.9%), of white ethnicity (85.7%), MSM (77.4%) with a median (IQR) age of 53 (47, 59) years. The majority of participants (91.4%) had an undetectable viral load (<50 copies/mL). The median (IQR) nadir CD4^+^ T-cell count and years since HIV diagnosis were 205 (108, 310) cells/μL and 13.2 (7.7, 20.4), respectively. Current smoking was reported by 23.9% of participants, current recreational drug use by 27.2% and a history of IDU by 9.5%.

**TABLE 1:** Demographic, lifestyle and HIV-related characteristics of POPPY participants with HIV (*n* = 788).

Characteristic *n* (%) or median (IQR)	Total cohort (*n* = 788)
*Demographic*	
Age (years)	53 (47–59)
Gender	
Male	677 (85.9)
Female	111 (14.1)
Ethnicity	
Black-African	113 (14.3)
White	675 (85.7)
*Lifestyle*	
Sexual orientation	
MSM	610 (77.4)
Heterosexual	178 (22.6)
BMI (kg/m^2^)	25.5 (23.2–28.2)
Smoking status	
Non-smoker	325 (41.2)
Ex-smoker	275 (34.9)
Smoker	188 (23.9)
Alcohol use	
No alcohol use	62 (7.9)
Ex alcohol use	91 (11.6)
Current alcohol use	635 (80.6)
Current recreational drug use (no vs. yes)	214 (27.2)
History of injection drug use (no vs. yes)	75 (9.5)
*HIV-related*	
Undetectable viral load	720 (91.4)
Nadir CD4+ count (cells/μl)	205 (108–310)
Duration of HIV (years)	13.2 (7.7–20.4)
On ART	769 (97.6)
Prior AIDS (no vs. yes)	230 (29.2)

### Patterns of multimorbidity

**FIGURE 1:**
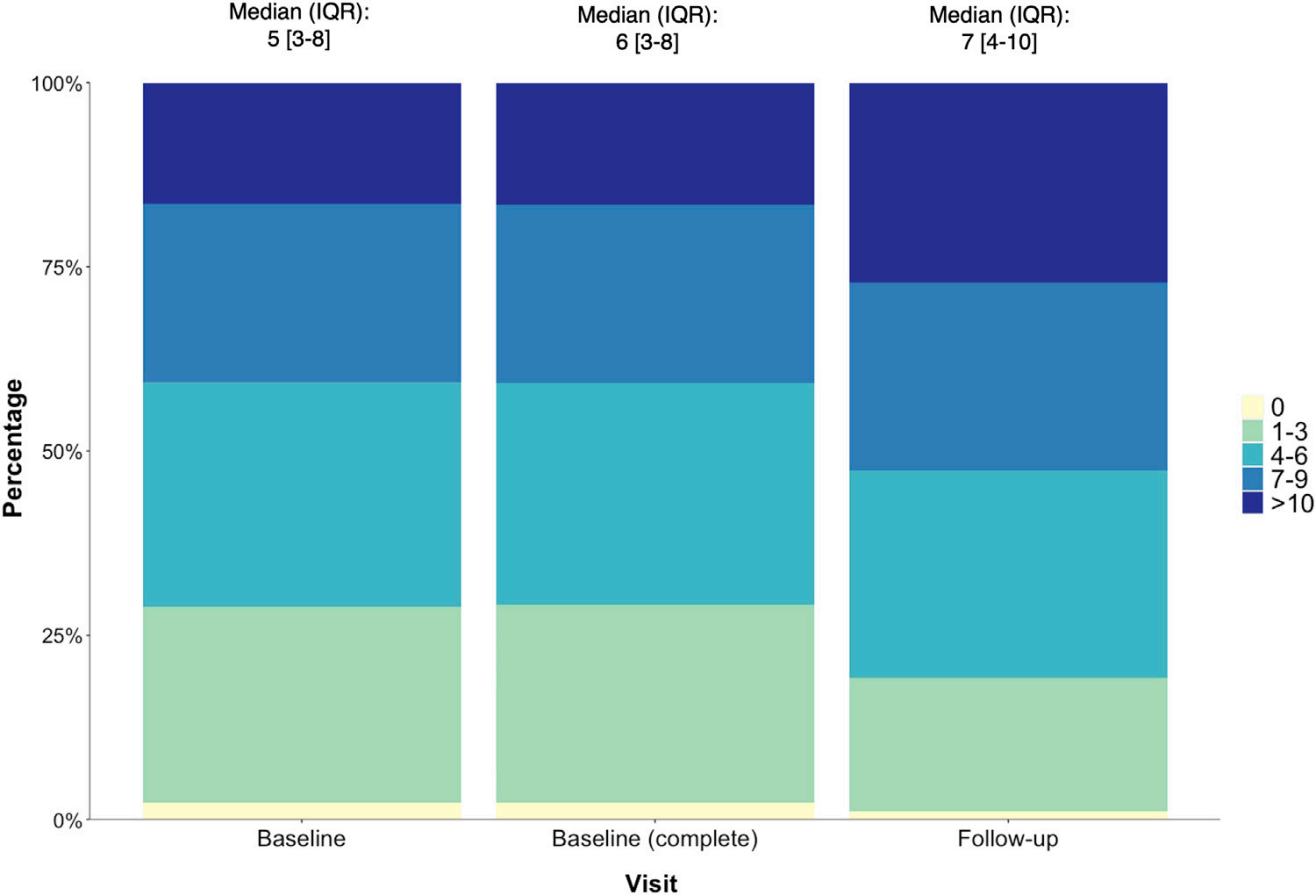
The number (%) of comorbidities reported by POPPY participants with HIV at baseline (*n* = 1,073), baseline (returned for follow-up, *n* = 788) and follow-up (*n* = 788), with the median (IQR) number of comorbidities per individual also reported.

Among the 788 participants, the prevalence of comorbidities ranged from 1% (pancreatitis) to 42% (gonorrhoea) (Supplementary Table S3). Other prevalent comorbidites included dyslipidemia (31%), clinical depression (30%) and syphilis (30%). In addition, 97.7% of all participants reported ≥ 1 comorbidity (median [IQR] per individual: 6 [3–8]) at baseline. At follow-up, 98.9% reported ≥1 comorbidity with a median [IQR] of 7 [4–10] comorbidities per individual (Figure 1). Most comorbidities demonstrated a similar small increase in prevalence, with the exception of the following conditions: joint/back pain (increased by 16%), chest infections (9%), osteopenia/osteoporosis (6.6%) and eye problems (6%) which had increased over the 3–5 year period (Supplementary Table S3).

Five common patterns were identified, accounting for 21.1% of the total variance in the 73 comorbidities. The comorbidities with a correlation (loading) ≥ 0.4 with each distinguished pattern are reported in Table 2. Three patterns were previously identified (De Francesco et al., 2018): ‘*CVDs*’ (hypertension, coronary artery bypass(CABG)/percutaneous transluminal coronary angioplasty (PCTA)), ‘*STDs*’ (gonorrhoea, *chlamydia*, syphilis, *Lymphogranuloma venereum* and herpes simplex virus) and ‘*Cancer*’ (haematological cancer and solid organ cancer). In addition, two novel patterns were identified: ‘*Neurometabolic*’ (pancreatic insufficiency, peripheral neuropathy, *Pneumocystis* pneumonia, pruritis, type II diabetes and hypothyroidism) and ‘*Mental-gastro-joint*’ pattern (clinical depression, joint inflammation/arthritis, pancreatitis, persistent bowel disorders and joint replacement).

**TABLE 2:** Patterns identified using principal component analysis in all POPPY participants with HIV (*n* = 1,073).

PC (% of variance explained)	Label	Comorbidities with correlation ≥ 0.4 (correlation with PC)
1 (6.5%)	CVD	Hypertension (0.76), CABG/PCTA (0.68), MI (0.68), Dyslipidemia (0.66), IHD (0.63), Heart failure (0.51)
2 (4.4%)	STDs	Gonorrhea (0.81), *Chlamydia* (0.66), Syphilis (0.63), LGV (0.61), HSV (0.48)
3 (3.7%)	Neurometabolic	Pancreatic insufficiency (0.66), Peripheral neuropathy (0.54), PCP (0.43), Pruritis (0.43), Type II diabetes (0.42), Hypothyroidism (0.40)
4 (3.5%)	Cancer	Haematological cancer (0.89), Solid organ cancer (0.80)
5 (3.0%)	Mental-gastro-joint	Clinical depression (0.77), Joint inflammation/arthritis (0.52), Pancreatitis (0.51), Persistent bowel disorders (0.48), Joint replacement (0.43)

CVD, Cardiovascular disease; CABG/PCTA, Coronary artery bypass/percutaneous transluminal coronary angioplasty; MI, Myocardial infarction; IHD, Ischemic heart disease; STD, Sexually transmitted diseases; LGV, Lymphogranuloma venereum; HSV, Herpes simplex virus; PCP, Pneumocystis pneumonia.

### Burden of multimorbidity patterns over time

**FIGURE 2:**
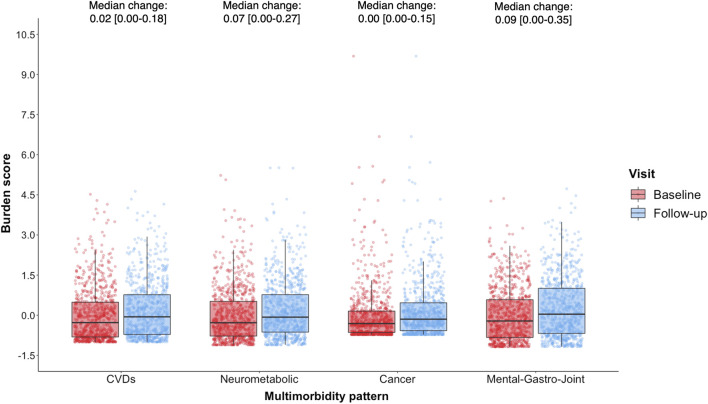
Distribution of multimorbidity burden z-scores at baseline (red) and follow-up (blue) among POPPY participants with HIV (*n* = 788), with median change [interquartile range; IQR] also reported.

The distribution of burden z-scores for the patterns at baseline and follow-up are shown in Figure 2. The highest median (IQR) z-score was reported in the *Mental-gastro-joint* pattern at both baseline (−0.21 [−0.83,0.58]) and follow-up (0.04 [−0.68,1.01]), with the largest median (IQR) change also observed for this pattern (0.09 [0.00,0.35]). There was considerable variation in the median distribution of both the *CVD* (baseline: −0.28 [−0.81,0.49]; follow-up: −0.06 [−0.72,0.77]) and *Neurometabolic* (baseline: −0.28 [−0.78,0.52]; follow-up: −0.07 [−0.63,0.77]) z-scores at baseline and follow-up. In contrast, the majority of participants reported extremely low z-scores in the *Cancer* pattern at both time points, with a very small change in this z-score: 0.00 [0.00–0.15].

### Risk factors associated with baseline multimorbidity burden

A longer duration since HIV diagnosis (range: 0.09–0.17, all *p* < 0.01) and a prior AIDS diagnosis (range: 0.19–0.83, all *p* < 0.01) were significantly associated with higher z-scores (1 standard deviation (SD) higher score) across all patterns at baseline (Table 3). Older age (0.37 [0.30, 0.44]), being male (0.35 [0.07, 0.63]) and a higher BMI (0.03 [0.01, 0.05] per 1-kg/m^2^ increment) were independently associated with higher *CVD* z-scores. In contrast, participants of white ethnicity had a lower z-score compared to those of Black-African ethnicity (−0.33 [−0.62, −0.05]). Similarly, older age (0.16 [0.09, 0.22]), sex between men (0.29 [0.05, 0.54], a higher BMI (0.02 [0.01, 0.03] per 1-kg/m^2^ increment) and current recreational drug use (0.14 [0.00, 0.28]) were associated with higher *Neurometabolic* z-scores. Older age (0.16 [0.08, 0.23]) was also associated with higher *Cancer* z-scores. Men had lower *Mental-gastro-joint* z-scores compared to women at baseline (−0.44 [−0.73, −0.15]). On the other hand, sex between men (0.32 [0.04, 0.59]), a higher BMI (0.02 [0.01, 0.04] per 1-kg/m^2^ increment), current smoking (0.25 [0.07, 0.42]), and a history of IDU (0.46 [0.23, 0.68]) were associated with higher *Mental-gastro-joint* z-scores. Furthermore, in the sub-analysis (*n* = 770), CD4:CD8 ratio was not significantly associated with any of the patterns at baseline (Supplementary Table S4).

**TABLE 3:** The relative contributions of demographic, lifestyle and clinical factors to the burden of multimorbidity patterns at baseline (*n* = 788) assessed using multivariable linear regression models, with regression coefficients (95% confidence intervals) and associated *p*-value reported.

**Risk factor**	**Baseline burden z-scores**			
	**CVD**	**Neurometabolic**	**Cancer**	**Mental-gastro-joint**
Age (per 10-year)	0.37 (0.30, 0.44) *p* < 0.001	0.16 (0.09, 0.22) *p* < 0.001	0.16 (0.08, 0.23) *p* < 0.001	0.04 (−0.03, 0.11) *p* = 0.24
Gender (male vs. female)	0.35 (0.07, 0.63) *p* = 0.01	−0.13 (−0.39, 0.13) *p* = 0.32	0.12 (−0.19, 0.43) *p* = 0.45	−0.44 (−0.73, −0.15) *p* < 0.001
Race (White vs. black-African)	−0.33 (−0.62, −0.05) *p* = 0.02	−0.21 (−0.47, 0.06) *p* = 0.13	0.11 (−0.21, 0.42) *p* = 0.51	0.24 (−0.06, 0.54) *p* = 0.12
Sexuality (MSM vs. heterosexual)	0.06 (−0.20, 0.32) *p* = 0.64	0.29 (0.05, 0.54) *p* = 0.02	0.04 (−0.25, 0.34) *p* = 0.77	0.32 (0.04, 0.59) *p* = 0.02
BMI (per 1-kg/m^2^)	0.03 (0.01, 0.05) *p* < 0.001	0.02 (0.01, 0.03) *p* = 0.01	0 (−0.02, 0.01) *p* = 0.80	0.02 (0.01, 0.04) *p* < 0.001
Smoking status				
Non-smoker vs. ex-smoker	0.08 (−0.06, 0.23) *p* = 0.27	0.03 (−0.11, 0.16) *p* = 0.70	0.04 (−0.12, 0.20) *p* = 0.65	0.11 (−0.05, 0.26) *p* = 0.17
Non-smoker vs. smoker	0.03 (−0.13, 0.20) *p* = 0.69	0.02 (−0.14, 0.18) *p* = 0.79	0.05 (−0.14, 0.23) *p* = 0.62	0.25 (0.07, 0.42) *p* = 0.01
Alcohol use				
No vs. ex alcohol use	−0.06 (−0.36, 0.23) *p* = 0.67	0.25 (−0.02, 0.53) *p* = 0.07	0.04 (−0.28, 0.37) *p* = 0.80	0.04 (−0.27, 0.35) *p* = 0.81
No vs. current alcohol use	0.02 (−0.22, 0.27) *p* = 0.87	0.12 (−0.11, 0.35) *p* = 0.32	0 (−0.27, 0.28) *p* = 0.97	−0.13 (−0.39, 0.13) *p* = 0.33
Current recreational drugs (yes vs. no)	−0.02 (−0.16, 0.13) *p* = 0.83	0.14 (0.00, 0.28) *p* = 0.05	0.04 (−0.13, 0.20) *p* = 0.67	0.15 (−0.01, 0.30) *p* = 0.06
History of IDU (yes vs. no)	0 (−0.22, 0.21) *p* = 0.98	0.14 (−0.06, 0.34) *p* = 0.18	−0.01 (−0.25, 0.23) *p* = 0.93	0.46 (0.23, 0.68) *p* < 0.001
Nadir CD4+ T-cell count (per 100 cells/μl)	0.03 (−0.01, 0.06) *p* = 0.18	−0.01 (−0.05, 0.02) *p* = 0.55	0 (−0.05, 0.04) *p* = 0.87	0.01 (−0.03, 0.05) *p* = 0.47
Years since HIV diagnosis (per 5-year)	0.13 (0.09, 0.18) *p* < 0.001	0.16 (0.12, 0.20) *p* < 0.001	0.09 (0.04, 0.13) *p* < 0.001	0.17 (0.12, 0.21) *p* < 0.001
Prior AIDS event (yes vs. no)	0.19 (0.05, 0.33) *p* = 0.01	0.83 (0.69, 0.96) *p* < 0.001	0.35 (0.19, 0.50) *p* < 0.001	0.27 (0.12, 0.41) *p* < 0.001

### Risk factors associated with an increase in multimorbidity burden over time

A longer time since HIV diagnosis was significantly associated with an increase in z-scores (1 SD) across all patterns (range: 0.02–0.03; all *p* < 0.01) (Table 4). None of the other risk factors were significantly associated with an increase in *Cancer or Mental-gastro-joint* z-scores. In contrast, older age per 10-year increment (0.04 [0.01, 0.06], *p <* 0.001) and a higher BMI (0.01 [0.01, 0.02] per 1-kg/m^2^, *p* = 0.02) were independently associated with an increase in *CVD* z-scores over time. Similarly, older age (0.03 [0.01, 0.05], *p =* 0.01) and a higher BMI (0.01 [0.00, 0.01] per 1-kg/m^2^, *p =* 0.01) were significantly associated with an increase in *Neurometabolic* z-scores. Furthermore, in the sub-analysis, CD4:CD8 ratio was not found to be associated with an increase in scores across any of the patterns over time (Supplementary Table S5).

**TABLE 4:** The relative contributions of demographic, lifestyle and clinical factors to an increase in burden of multimorbidity patterns (*n* = 788) assessed using multivariable linear regression models, with regression coefficients (95% confidence intervals) and associated *p*-value reported.

**Risk factor**	**Change in burden z-scores**			
	**CVD**	**Neurometabolic**	**Cancer**	**Mental-gastro-joint**
Age (per 10-year)	0.04 (0.01, 0.06) *p* < 0.001	0.03 (0.01, 0.05) *p* = 0.01	0.02 (−0.01, 0.05) *p* = 0.22	0.00 (−0.03, 0.03) *p* = 0.89
Gender (male vs. female)	0.02 (−0.08, 0.13) *p* = 0.69	−0.04 (−0.13, 0.05) *p* = 0.36	0.01 (−0.13, 0.14) *p* = 0.92	−0.04 (−0.15, 0.08) *p* = 0.55
Race (White vs. black-African)	0.03 (−0.08, 0.13) *p* = 0.64	0.08 (−0.02, 0.17) *p* = 0.11	0.01 (−0.14, 0.15) *p* = 0.93	0.03 (−0.09, 0.15) *p* = 0.64
Sexuality (MSM vs. heterosexual)	−0.02 (−0.12, 0.08) *p* = 0.75	−0.02 (−0.10, 0.07) *p* = 0.72	0.08 (−0.05, 0.21) *p* = 0.23	0.01 (−0.10, 0.12) *p* = 0.89
BMI (per 1-kg/m^2^)	0.01 (0.01, 0.02) *p* < 0.001	0.01 (0.00, 0.01) *p* = 0.01	0.00 (−0.01, 0.01) *p* = 0.66	0.00 (−0.01, 0.01) *p* = 0.85
Smoking status				
Non-smoker vs. ex-smoker	0.01 (−0.04, 0.07) *p* = 0.63	−0.01 (−0.06, 0.04) *p* = 0.68	0.02 (−0.06, 0.09) *p* = 0.66	−0.02 (−0.08, 0.04) *p* = 0.49
Non-smoker vs. smoker	0.01 (−0.05, 0.08) *p* = 0.66	0.00 (−0.05, 0.06) *p* = 0.95	−0.03 (−0.12, 0.05) *p* = 0.42	0.03 (−0.04, 0.10) *p* = 0.35
Alcohol use				
No vs. ex alcohol use	0.05 (−0.06, 0.16) *p* = 0.39	0.03 (−0.07, 0.13) *p* = 0.58	0.02 (−0.13, 0.17) *p* = 0.79	0.01 (−0.11, 0.14) *p* = 0.84
No vs. current alcohol use	0.03 (−0.06, 0.13) *p* = 0.48	0.02 (−0.06, 0.10) *p* = 0.6	0.05 (−0.07, 0.17) *p* = 0.45	0 (−0.10, 0.10) *p* = 0.99
Current recreational drugs (yes vs. no)	0.03 (−0.03, 0.09) *p* = 0.28	0.02 (−0.03, 0.07) *p* = 0.36	−0.02 (−0.09, 0.05) *p* = 0.58	−0.01 (−0.08, 0.05) *p* = 0.64
History of IDU (yes vs. no)	0.02 (−0.06, 0.10) *p* = 0.57	0.03 (−0.04, 0.10) *p* = 0.45	0.06 (−0.05, 0.17) *p* = 0.27	0.09 (−0.00, 0.18) *p* = 0.06
Nadir CD4+ T-cell count (per 100 cells/μl)	0.01 (−0.00, 0.03) *p* = 0.12	0.00 (−0.01, 0.02) *p* = 0.44	0.01 (−0.01, 0.03) *p* = 0.50	0.01 (−0.00, 0.03) *p* = 0.13
Years since HIV diagnosis (per 5-year)	0.02 (0.01, 0.04) *p* < 0.001	0.02 (0.01, 0.04) *p* < 0.001	0.03 (0.01, 0.06) *p* < 0.001	0.03 (0.01, 0.05) *p* < 0.001
Prior AIDS event (yes vs. no)	−0.02 (−0.07, 0.03) *p* = 0.46	0.01 (−0.04, 0.05) *p* = 0.76	0.01 (−0.06, 0.08) *p* = 0.70	−0.02 (−0.08, 0.04) *p* = 0.52

## Discussion

This study identified five common multimorbidity patterns in a cohort of people living with HIV receiving clinical care in the United Kingdom and Ireland and found that their burden has increased over a 3–5 year period. We also characterised the relative contributions of HIV- and non-HIV-related risk factors, and report key modifiable (e.g., BMI and recreational/injection drug use) and non-modifiable (e.g., age and time since HIV diagnosis) factors associated with the rise in multimorbidity burden.

Existing studies on multimorbidity patterns among people with HIV are scarce and vary considerably in terms of their methodological approach. In particular, a small number of comorbidities (typically 14–24) are considered, which may not reflect the wider spectrum of health conditions typically reported in people with HIV (Morales et al., 2022). We re-identified patterns using a wide spectrum of comorbidities reflecting the complexity of multimorbidity in this population. In doing so, two novel patterns were identified (*Neurometabolic* and *Mental-gastro-joint*), along with three patterns (*CVDs, STDs* and *Cancer*) that were previously identified among this cohort (De Francesco et al., 2018), and are consistent with those reported by other studies in people with HIV (Goulet et al., 2005; Yang et al., 2021). However, it is difficult to compare our results with those of previous studies due to differences in statistical methods, data sources, study populations and the comorbidities included. In both general populations and people with HIV, pancreatic insufficiency, hypothyroidism, pruritis and peripheral neuropathy have been linked to type 2 diabetes (Evans et al., 2012; Piciucchi et al., 2015; Ogbonna and Ezeani, 2019; Pillay et al., 2020; Stefaniak et al., 2021; Kuka et al., 2022). However, the potential shared underlying biological pathways associated with these comorbidities merits further investigation. For example, exploring whether the comorbidities in the *Neurometabolic* pattern are more likely to co-occur due to contributions from HIV-mediated inflammatory processes such as persistent inflammation. The biological link between mental health and joint disorders has been supported by other clinical studies that suggest a bidirectional relationship in which local and systemic inflammation play an important role (Lu et al., 2019; Nikiphorou et al., 2019; Lwin et al., 2020). HIV-associated systemic inflammation may also explain the co-occurrence of gastrointestinal disorders within this pattern (Brenchley et al., 2008).

In the present study, we found that the contributions of HIV- and non-HIV-related risk factors to the burden at baseline and over time varied across patterns. With the demographic/lifestyle factors, older age and a higher BMI were associated with higher *CVDs*, *Neurometabolic* and *Cancer* burden at baseline. However, these factors were only associated with an increase in *CVDs* and *Neurometabolic* burden over time. With the behavioural factors, history of IDU and current recreational drug use were associated with higher *Mental-gastro-joint* and *Neurometabolic* burden at baseline, respectively, which is consistent with previous studies linking IDU/recreational drug use to the development of mood disorders and metabolic complications(Henry, 2000; Williams et al., 2017). However, none of the behavioural factors were identified as predictors for an increase in burden of any of the patterns. One possible explanation for this may be that a longer follow-up period may be required to reveal their impact over time. Alternatively, this could reflect reverse causation as the majority of comorbidities preceded the assessment of the behavioural factors, and thus people may already have made changes to their lifestyles after a previous diagnosis of a comorbidity. In terms of the HIV-related factors, a prior AIDS diagnosis and a longer duration since HIV diagnosis were associated with higher scores across all the patterns at baseline. However, only the latter was identified as a predictor for an increase in burden of these patterns over time. Nevertheless, these findings suggest that biological effects of HIV infection, including sustained immune activation and chronic inflammation, may play a central role in the burden of multimorbidity among people with HIV.

To our knowledge, this is the first study to assess the burden of multimorbidity patterns, and the relative contributions of risk factors, over time. However, there are some limitations to our study that need to be considered. First, there is no uniform list of comorbidities to define multimorbidity among people with HIV, so the list considered here may be debated. However, we used a large list to ensure we could capture the diverse/wide spectrum of comorbidities typically reported in this population. Second, data on comorbidities were collected using self-reported medical history, and were thus subject to recall bias. However, we ensured that, where possible, information was collected/validated using healthcare utilisation and concomitant medication data. Third, multimorbidity burden over time was examined using patterns identified at baseline. We acknowledge that the loadings of comorbidities may have changed after a follow-up period of 3–5 years, but examining scores based on baseline patterns allowed us to compare both time points. Fourth, the exploration of alternative methodologies such as Actionable Explainable Artificial intelligence (AxAI) (a machine learning approach)(Saranti et al., 2022), as well as a longer follow-up period, may have been advantageous to assess the change in multimorbidity burden and to elucidate the causal link with risk factors. The POPPY study has two additional study visits planned for data collection, which will allow us to reexamine the burden of multimorbidity over a longer follow-up period. Finally, the POPPY cohort is predominantly men of white ethnicity (representative of the UK HIV population seen in clinical care); therefore, our findings are less generalizable to cohorts in different HIV settings, i.e., populations that are predominately women and/or of non-white ethnicity.

## Conclusion

This study represents one of the first efforts to describe multimorbidity burden over time among people with HIV. Our work highlights an increasing need for the development of targeted interventions, particularly focusing on modifiable risk factors, and guidelines to address the complexities and management of multimorbidity in the context of HIV. Our findings also highlight the need for further work to elucidate the role of HIV-mediated immunological pathways (using biomarker data) to the burden of multimorbidity. Future planned work will also explore the impact of multimorbidity burden on health/treatment outcomes over time.

## Data Availability

The original contributions presented in the study are included in the article/Supplementary Material, further inquiries can be directed to the corresponding author.
