# Supplementary material for: Changes in multimorbidity burden over a 3–5 year period among people with HIV
Source: Front Syst Biol. 2023 Feb 27;3:1136999. doi: 10.3389/fsysb.2023.1136999 (PMC12341977; doi:10.3389/fsysb.2023.1136999)
Supplement: Supplementary file 1 [file Table1.DOCX]

Changes in multimorbidity burden over a 3-5 year period among people with HIV

**SUPPLEMENTARY** **DATA**

(in order of where first listed)

**Supplementary Table 1.** The final list of 73 individual comorbidities included in the analyses reported in this thesis

| Organ system/ pathogenic group | Comorbidity | Organ system/ pathogenic group | Comorbidity |
| --- | --- | --- | --- |
| AIDS event | Tuberculosis | **Hepatitis** | Hepatitis A |
|  | Cytomegalovirus |  | Hepatitis B |
|  | Pneumocystis pneumonia |  | Hepatitis C |
|  | Kaposi’s sarcoma | **Gastrointestinal disorders** | Persistent bowel disorder |
|  | AIDS-related cancers |  | Hernia |
|  | Other AIDS events |  | Gastroesophageal reflux disease |
| Infections | Varicella zoster virus |  | Pancreatic insufficiency |
|  | Fungal infection |  | Liver diseases |
| Endocrine diseases | Type 2 diabetes | **Genitourinary disorders** | Renal problems |
|  | Lipodystrophy/lipoatrophy |  | Urinary incontinence |
|  | Dyslipidemia |  | Erectile dysfunction |
|  | Hypothyroidism |  | Prostate dysfunction |
|  | Pancreatitis |  | Kidney stones |
|  | Hypogonadism/ low testosterone |  | Urinary tract infection |
| Mental health problems | Clinically diagnosed depression | **Bone and joint disorders** | Joint inflammation/arthritis |
|  | Depressive symptoms |  | Joint replacement |
|  | Anxiety/ panic attacks |  | Osteopenia/osteoporosis |
|  | Sleeping problems |  | Joint/back pain |
|  | Psychosis | **Sexually transmitted disorders** | Syphilis |
| Nervous system problems | Dizziness/vertigo |  | Gonorrhea |
|  | Loss of consciousness |  | Chlamydia |
|  | Encephalitis |  | Lymphogranuloma venereum |
|  | Epilepsy |  | Human papilloma virus |
|  | Peripheral neuropathy |  | Herpes simplex virus |
|  | Migraine/ headaches | **Respiratory diseases** | Asthma/bronchitis/chronic obstructive pulmonary disease, bronchiectasis |
| Haematological disorders | Anaemia |  | Pneumonia |
| Cardiovascular diseases | Myocardial infarction |  | Chest infection |
|  | Angina pectoris |  | Hayfever/allergy |
|  | Peripheral vascular disease |  | Chronic cough |
|  | Hypertension | **Skin conditions** | Psoriasis |
|  | Transient ischemic attack |  | Eczema/dermatitis |
|  | Coronary artery bypass grafting |  | Pruritus |
|  | Heart failure | **Ear dysfunction** | Ear dysfunction |
|  | Ischaemic heart disease | **Eye problem** | Eye problem |
|  | Arrhythmia | **Vitamin D deficiency** | Vitamin D deficiency |

**Supplementary Table 2.** Descriptive comparison between the included (n=788) and excluded (n=285) sample

| **Characteristic  n (%) or median (IQR)** | **Included cohort**  **(n=788)** | **Excluded cohort**  **(n=285)** | p† |
| --- | --- | --- | --- |
| Age (years) | 53 (47 – 59) | 51 (44 – 59) | *P=*0.05 |
| Gender |  |  | *P*=0.26 |
| Male | 677 (85.9) | 237 (83.2) |  |
| Female | 111 (14.1) | 48 (16.8) |  |
| Ethnicity |  |  | *P*=0.02 |
| Black-African | 113 (14.3) | 58 (20.4) |  |
| White | 675 (85.7) | 227 (79.7) |  |
| Sexual orientation |  |  | *P*=0.08 |
| MSM | 610 (77.4) | 206 (72.3) |  |
| Heterosexual | 178 (22.6) | 79 (27.7) |  |
| Smoking status |  | 82 (28.0) | *P*=0.29 |
| Non-smoker | 325 (41.2) | 114 (40.0) |  |
| Ex-smoker | 275 (34.9) | 90 (31.6) |  |
| Smoker | 188 (23.9) | 81 (28.4) |  |
| Alcohol use |  | 227 (77.5) | *P*=0.57 |
| No alcohol use | 62 (7.9) | 28 (9.8) |  |
| Ex alcohol use | 91 (11.6) | 34 (11.9) |  |
| Current alcohol use | 635 (80.6) | 223 (78.3) |  |
| Current recreational drug use | 214 (27.2) | 93 (32.6) | *P*=0.08 |
| History of injection drug use | 75 (9.5) | 37 (13.0) | *P*=0.11 |
| Undetectable viral load | 720 (91.4) | 260 (91.2) | *P*=0.94 |
| On ART | 769 (97.6) | 277 (97.2) | *P*=0.72 |
| Prior AIDS (no vs. yes) | 230 (29.2) | 81 (28.4) | *P*=0.81 |
| ^†^p-value for between-cohort differences; calculated using chi^2^ (categorical variables) or Kruskal-Wallis (continuous variables) | | | |

**Supplementary Figure 1. Principial component analysis (PCA) loadings of comorbidities for the Cardiovascular disease pattern**

**
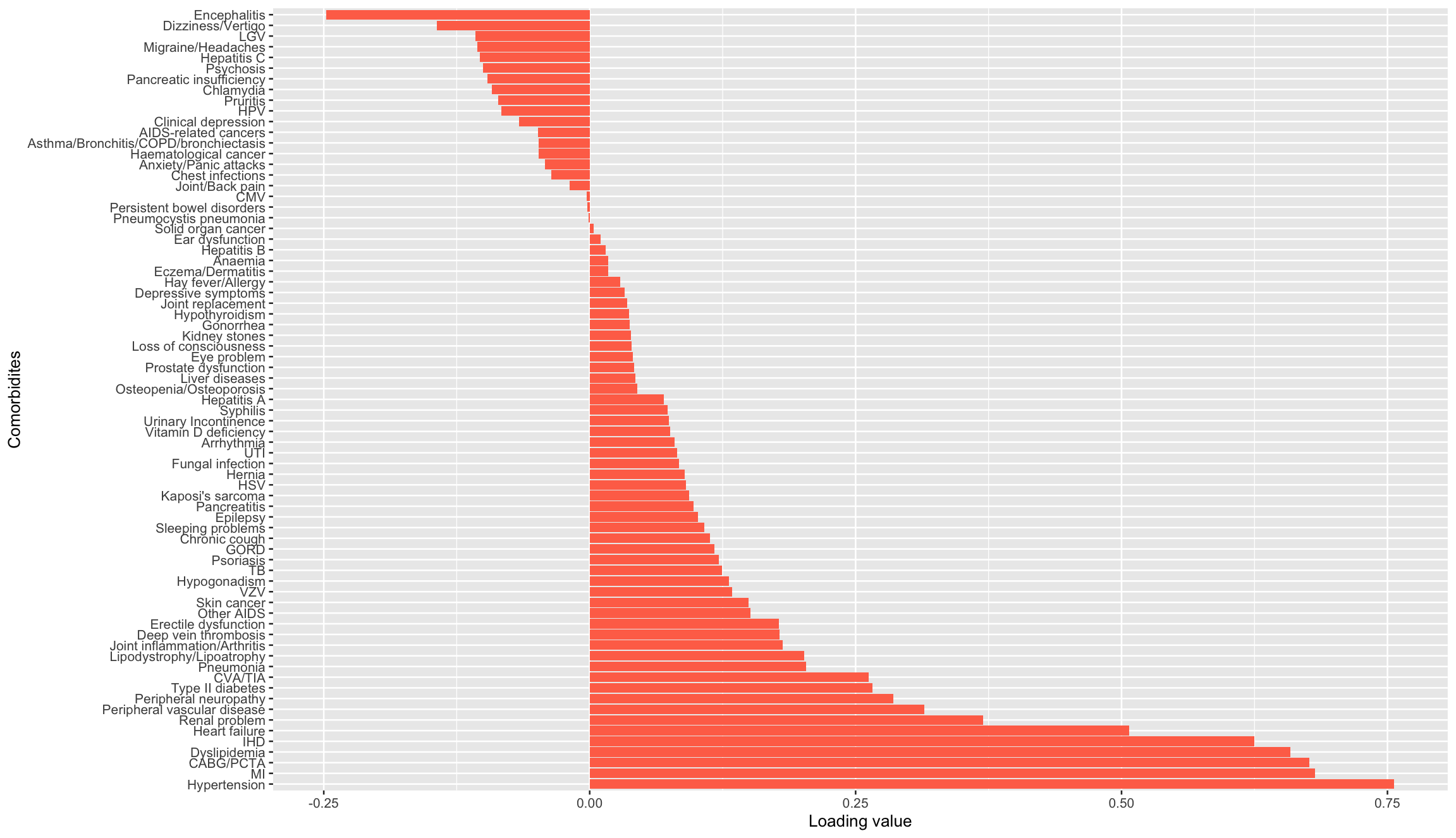
**

**Supplementary Figure 2. Principial component analysis (PCA) loadings of comorbidities for the Neurometabolic pattern**

**
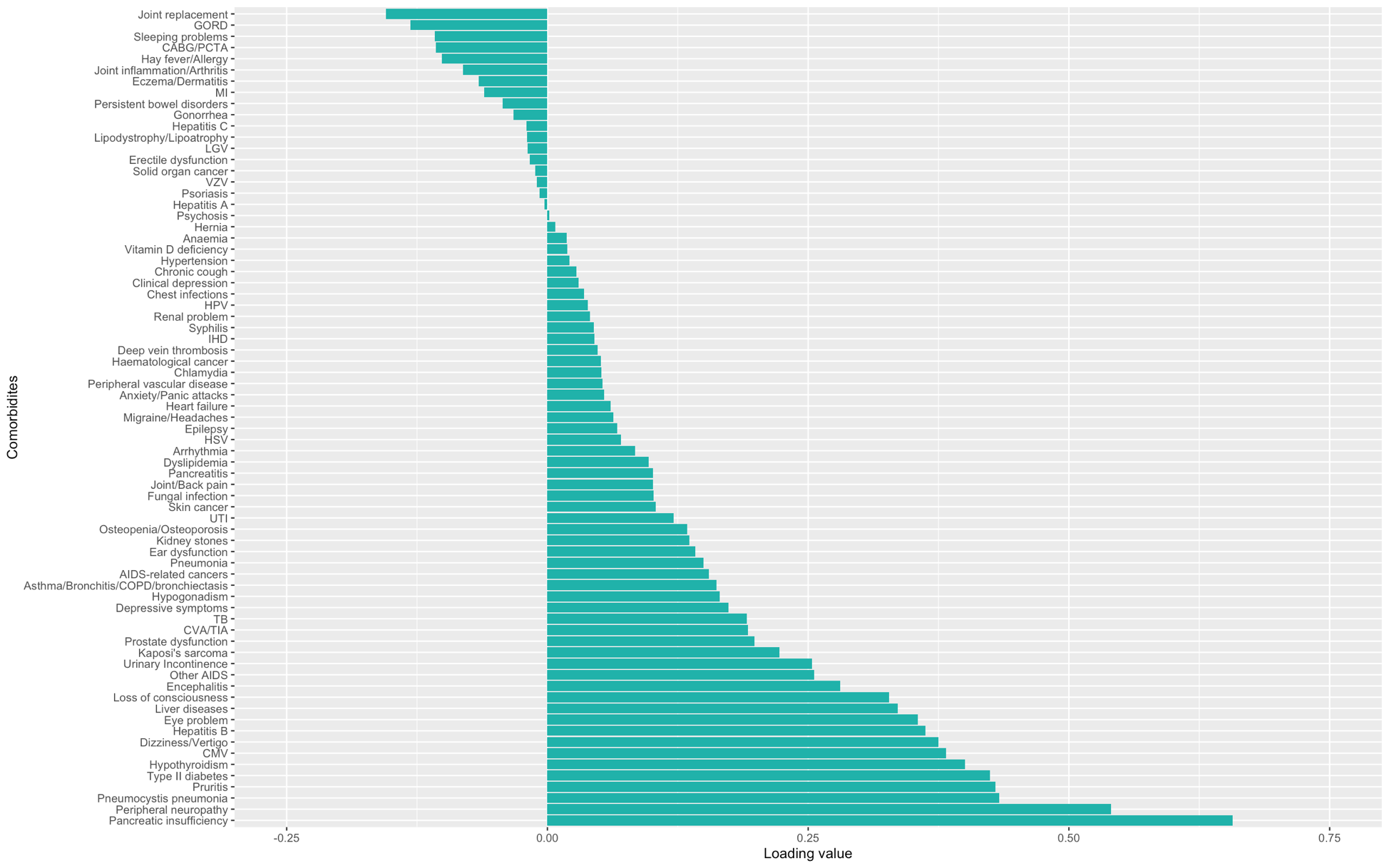
**

**Supplementary Figure 3. Principial component analysis (PCA) loadings of comorbidities for the Cancer pattern**

**
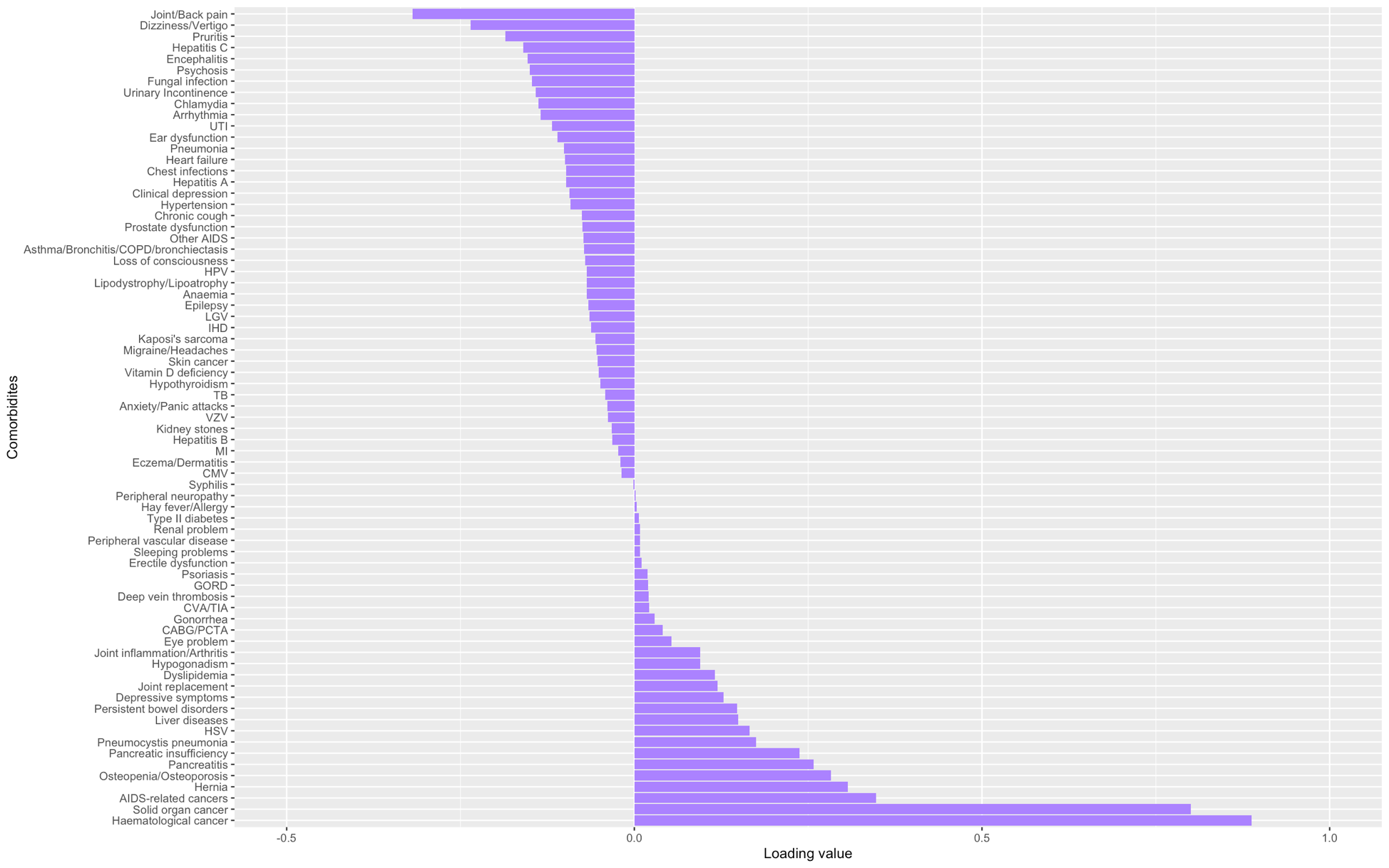
**

**Supplementary Figure 4. Principial component analysis (PCA) loadings of comorbidities for the Mental-gastro-joint pattern**

**
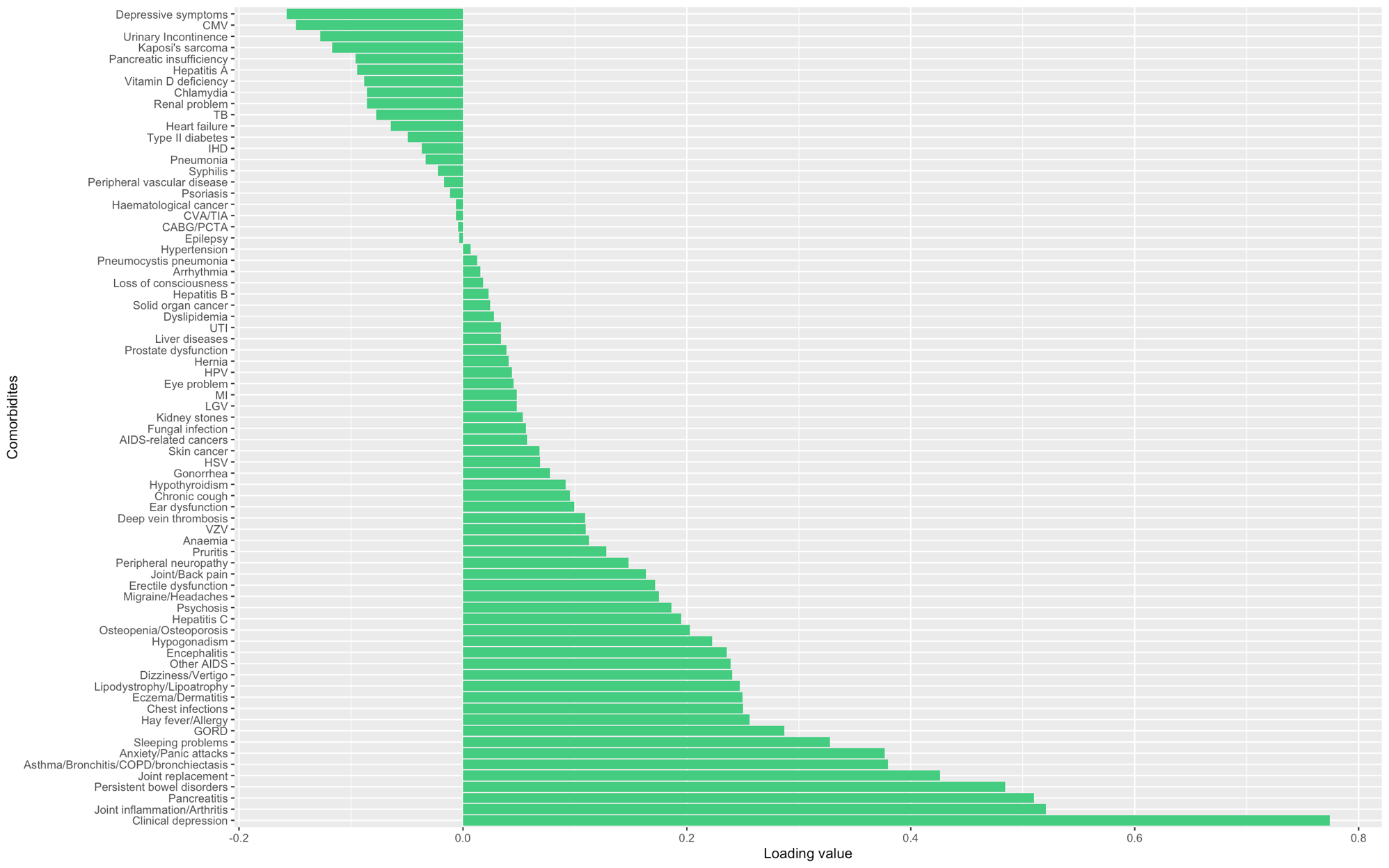
**

**Supplementary Table 3**. The prevalence of comorbidities in POPPY participants with HIV (n=788) at baseline and follow-up (cumulative)

| Organ / pathogenic group | Comorbidity | Baseline prevalence  n (%) | Follow-up prevalence  n (%) | Organ / pathogenic group | Comorbidity | Baseline prevalence n (%) | Follow-up prevalence  n (%) |
| --- | --- | --- | --- | --- | --- | --- | --- |
| AIDS event | Tuberculosis | 59 (7.5) | 59 (7.5) | **Hepatitis** | Hepatitis A | 32 (4.1) | 32 (4.1) |
|  | Cytomegalovirus | 18 (2.3) | 18 (2.3) |  | Hepatitis B | 139 (17.6) | 140 (17.8) |
|  | Pneumocystis pneumonia | 70 (8.9) | 70 (8.9) |  | Hepatitis C | 77 (9.8) | 82 (10.4) |
|  | Kaposi’s sarcoma | 56 (7.1) | 56 (7.1) | **Gastrointestinal disorders** | Persistent bowel disorder | 74 (9.4) | 90 (11.4) |
|  | AIDS-related cancers | 15 (1.9) | 15 (1.9) |  | Hernia | 34 (4.3) | 48 (6.1) |
|  | Other AIDS events | 78 (9.9) | 78 (9.9) |  | Gastroesophageal reflux disease | 62 (7.9) | 83 (10.5) |
| Infections | Varicella zoster virus | 68 (8.6) | 79 (10) |  | Pancreatic insufficiency | 10 (1.3) | 13 (1.6) |
|  | Fungal infection | 38 (4.8) | 71 (9) |  | Liver diseases | 55 (7) | 62 (7.9) |
| Endocrine diseases | Type 2 diabetes | 44 (5.6) | 46 (5.8) | **Genitourinary disorders** | Renal problems | 36 (4.6) | 56 (7.1) |
|  | Lipodystrophy/lipoatrophy | 18 (2.3) | 33 (4.2) |  | Urinary incontinence | 33 (4.2) | 54 (6.9) |
|  | Dyslipidemia | 240 (30.5) | 266 (33.8) |  | Erectile dysfunction | 63 (8) | 77 (9.8) |
|  | Hypothyroidism | 21 (2.7) | 22 (2.8) |  | Prostate dysfunction | 28 (3.6) | 39 (4.9) |
|  | Pancreatitis | 10 (1.3) | 12 (1.5) |  | Kidney stones | 24 (3) | 36 (4.6) |
|  | Hypogonadism/ low testosterone | 29 (3.7) | 31 (3.9) |  | Urinary tract infection | 23 (2.9) | 44 (5.6) |
| Mental health problems | Clinically diagnosed depression | 238 (30.2) | 264 (33.5) | **Bone and joint disorders** | Joint inflammation/arthritis | 149 (18.9) | 177 (22.5) |
|  | Depressive symptoms | 55 (7) | 84 (10.7) |  | Joint replacement | 15 (1.9) | 19 (2.4) |
|  | Anxiety/ panic attacks | 62 (7.9) | 85 (10.8) |  | Osteopenia/osteoporosis | 57 (7.2) | 109 (13.8) |
|  | Sleeping problems | 56 (7.1) | 75 (9.5) |  | Joint/back pain | 146 (18.5) | 273 (34.6) |
|  | Psychosis | 16 (2) | 18 (2.3) | **Sexually transmitted disorders** | Syphilis | 234 (29.7) | 247 (31.3) |
| Nervous system problems | Dizziness/vertigo | 85 (10.8) | 110 (14) |  | Gonorrhea | 334 (42.4) | 348 (44.2) |
|  | Loss of consciousness | 19 (2.4) | 20 (2.5) |  | Chlamydia | 224 (28.4) | 225 (28.6) |
|  | Encephalitis | 13 (1.6) | 13 (1.6) |  | Lymphogranuloma venereum | 33 (4.2) | 36 (4.6) |
|  | Epilepsy | 31 (3.9) | 31 (3.9) |  | Human papilloma virus | 82 (10.4) | 91 (11.5) |
|  | Peripheral neuropathy | 168 (21.3) | 179 (22.7) |  | Herpes simplex virus | 115 (14.6) | 127 (16.1) |
|  | Migraine/ headaches | 44 (5.6) | 54 (6.9) | **Respiratory diseases** | Asthma/bronchitis/chronic obstructive pulmonary disease, bronchiectasis | 193 (24.5) | 209 (26.5) |
| Haematological disorders | Anaemia | 17 (2.2) | 25 (3.2) |  | Pneumonia | 44 (5.6) | 59 (7.5) |
|  | Deep vein thrombosis | 16 (2) | 20 (2.5) |  | Chest infection | 88 (11.2) | 159 (20.2) |
| Cardiovascular diseases | Myocardial infarction | 28 (3.6) | 32 (4.1) |  | Hayfever/allergy | 70 (8.9) | 79 (10) |
|  | Peripheral vascular disease | 13 (1.6) | 14 (1.8) |  | Chronic cough | 10 (1.3) | 20 (2.5) |
|  | Hypertension | 191 (24.2) | 218 (27.7) | **Skin conditions** | Psoriasis | 35 (4.4) | 43 (5.5) |
|  | Transient ischemic attack | 28 (3.6) | 33 (4.2) |  | Eczema/dermatitis | 85 (10.8) | 100 (12.7) |
|  | CABG/PTCA | 19 (2.4) | 22 (2.8) |  | Pruritus | 14 (1.8) | 21 (2.7) |
|  | Heart failure | 18 (2.3) | 22 (2.8) | **Cancer** | Skin cancer | 35 (4.4) | 52 (6.6) |
|  | Ischaemic heart disease / Angina | 34 (4.3) | 45 (5.7) |  | Haematological cancer | 10 (1.3) | 12 (1.5) |
|  | Arrhythmia | 30 (3.8) | 37 (4.7) |  | Solid organ cancer | 46 (5.8) | 60 (7.6) |
| Vitamin deficiency | Vitamin D deficiency | 18 (2.3) | 42 (5.3) | **Ear dysfunction** | Ear dysfunction | 54 (6.9) | 54 (6.9) |
|  |  |  |  | **Eye problem** | Eye problem | 68 (8.6) | 116 (14.7) |

**Supplementary Table 4.** The relative contributions of demographic, lifestyle and clinical factors to the burden of multimorbidity patterns in a subset of POPPY PLWH (n=770) at baseline assessed using multivariable linear regression models, with regression coefficients (95% confidence intervals) and associated p-value reported.

|  | **Baseline burden z-scores** | | | |
| --- | --- | --- | --- | --- |
| **Risk factor** | **CVD** | **Neurometabolic** | **Cancer** | **Mental-gastro-joint** |
| Age (per 10-year) | 0.37 (0.30, 0.44)  P<0.001 | 0.15 (0.09, 0.22)  P<0.001 | 0.15 (0.07, 0.22)  P<0.001 | 0.04 (-0.04, 0.11)  P=0.34 |
| Gender (female vs. male) | 0.37 (0.08, 0.66)  P=0.01 | -0.09 (-0.36, 0.18)  P=0.51 | 0.18 (-0.15, 0.50)  P=0.29 | -0.41 (-0.71, -0.10)  P=0.01 |
| Race (Black-African vs. White) | -0.36 (-0.65, -0.07)  P=0.02 | -0.23 (-0.51, 0.04)  P=0.1 | 0.08 (-0.25, 0.41)  P=0.62 | 0.24 (-0.07, 0.55)  P=0.14 |
| Sexuality (Heterosexual vs. MSM) | 0.07 (-0.20, 0.33)  P=0.61 | 0.29 (0.04, 0.54)  P=0.02 | 0.02 (-0.27, 0.32)  P=0.87 | 0.32 (0.04, 0.60)  P=0.03 |
| BMI (per 1-kg/m^2^) | 0.03 (0.02, 0.05)  P<0.001 | 0.02 (0.00, 0.03)  P=0.01 | 0 (-0.02, 0.02)  P=0.79 | 0.03 (0.01, 0.04)  P<0.001 |
| Smoking status |  |  |  |  |
| Non-smoker vs. ex-smoker | 0.07 (-0.07, 0.22)  P=0.32 | 0.02 (-0.12, 0.16)  P=0.81 | 0.02 (-0.14, 0.18)  P=0.8 | 0.1 (-0.05, 0.26)  P=0.19 |
| Non-smoker vs. smoker | 0.05 (-0.12, 0.22)  P=0.58 | 0.02 (-0.14, 0.19)  P=0.76 | 0.04 (-0.15, 0.23)  P=0.69 | 0.25 (0.07, 0.43)  P=0.01 |
| Alcohol use |  |  |  |  |
| No vs. ex alcohol use | -0.02 (-0.32, 0.29)  P=0.91 | 0.25 (-0.04, 0.54)  P=0.09 | 0.02 (-0.32, 0.35)  P=0.93 | 0.01 (-0.31, 0.33)  P=0.95 |
| No vs. current alcohol use | 0.06 (-0.20, 0.31)  P=0.67 | 0.12 (-0.12, 0.36)  P=0.34 | -0.02 (-0.31, 0.26)  P=0.87 | -0.14 (-0.41, 0.13)  P=0.31 |
| Current recreational drugs (no vs. yes) | -0.02 (-0.17, 0.12)  P=0.75 | 0.13 (-0.01, 0.27)  P=0.07 | 0.02 (-0.15, 0.19)  P=0.8 | 0.14 (-0.02, 0.29)  P=0.1 |
| History of IDU (no vs. yes) | 0.01 (-0.21, 0.22)  P=0.95 | 0.14 (-0.07, 0.34)  P=0.2 | -0.03 (-0.27, 0.21)  P=0.81 | 0.45 (0.22, 0.68)  P<0.001 |
| CD4:CD8 ratio | 0.05 (-0.10, 0.20)  P=0.5 | 0.07 (-0.07, 0.22)  P=0.32 | 0.07 (-0.10 , 0.24)  P=0.44 | 0.07 (-0.10 , 0.23)  P=0.43 |
| Nadir CD4+ T-cell count (per 100 cells/μl) | 0.02 (-0.02, 0.06)  P=0.24 | -0.02 (-0.05, 0.02)  P=0.4 | -0.01 (-0.05, 0.03)  P=0.7 | 0.01 (-0.03, 0.05)  P=0.65 |
| Years since HIV diagnosis (per 5-year) | 0.13 (0.09, 0.17)  P<0.001 | 0.17 (0.12, 0.21)  P<0.001 | 0.09 (0.04, 0.14)  P<0.001 | 0.17 (0.12, 0.21)  P<0.001 |
| Prior AIDS event (no vs. yes) | 0.18 (0.04, 0.31)  P=0.01 | 0.81 (0.68, 0.95)  P<0.001 | 0.35 (0.19, 0.51)  P<0.001 | 0.25 (0.11, 0.40)  P<0.001 |

**Supplementary Table 5.** The relative contributions of demographic, lifestyle and clinical factors to an increase in burden of multimorbidity patterns in a subset of POPPY PLWH (n=770) assessed using multivariable linear regression models, with regression coefficients (95% confidence intervals) and associated p-value reported.

|  | **Change in burden z-scores** | | | |
| --- | --- | --- | --- | --- |
| **Risk factor** | **CVD** | **Neurometabolic** | **Cancer** | **Mental-gastro-joint** |
| Age (per 10-year) | 0.04 (0.01, 0.07)  P<0.001 | 0.03 (0.01, 0.05)  P=0.01 | 0.02 (-0.01, 0.06)  P=0.21 | 0.00 (-0.03, 0.03)  P=0.88 |
| Gender (female vs. male) | 0.00 (-0.11, 0.11)  P=0.99 | -0.04 (-0.14, 0.05)  P=0.38 | 0.01 (-0.14, 0.15)  P=0.90 | -0.04 (-0.16, 0.08)  P=0.54 |
| Race (Black-African vs. White) | 0.03 (-0.08, 0.14)  P=0.60 | 0.08 (-0.02, 0.18)  P=0.11 | 0.00 (-0.14, 0.15)  P=0.96 | 0.05 (-0.07, 0.18)  P=0.38 |
| Sexuality (Heterosexual vs. MSM) | 0.00 (-0.10, 0.10)  P=0.99 | -0.01 (-0.10, 0.08)  P=0.81 | 0.07 (-0.06, 0.21)  P=0.27 | 0.02 (-0.09, 0.13)  P=0.77 |
| BMI (per 1-kg/m^2^) | 0.01 (0.01, 0.02)  P<0.001 | 0.01 (0.00, 0.01)  P=0.01 | 0.00 (-0.01, 0.01)  P=0.62 | 0.00 (-0.00, 0.01)  P=0.48 |
| Smoking status |  |  |  |  |
| Non-smoker vs. ex-smoker | 0.02 (-0.04, 0.07)  P=0.56 | -0.01 (-0.06, 0.04)  P=0.7 | 0.02 (-0.06, 0.09)  P=0.63 | -0.02 (-0.08, 0.04)  P=0.51 |
| Non-smoker vs. smoker | 0.02 (-0.04, 0.08)  P=0.55 | 0.00 (-0.05, 0.06)  P=0.87 | -0.03 (-0.12, 0.05)  P=0.42 | 0.04 (-0.03, 0.11)  P=0.27 |
| Alcohol use |  |  |  |  |
| No vs. ex alcohol use | 0.05 (-0.07, 0.17)  P=0.39 | 0.01 (-0.09, 0.11)  P=0.83 | 0.02 (-0.13, 0.17)  P=0.80 | -0.01 (-0.14, 0.12)  P=0.87 |
| No vs. current alcohol use | 0.03 (-0.07, 0.13)  P=0.53 | 0.01 (-0.07, 0.10)  P=0.74 | 0.05 (-0.08, 0.18)  P=0.45 | -0.01 (-0.12, 0.09)  P=0.80 |
| Current recreational drugs (no vs. yes) | 0.03 (-0.03, 0.09)  P=0.30 | 0.02 (-0.03, 0.07)  P=0.43 | -0.02 (-0.09, 0.06)  P=0.60 | -0.02 (-0.08, 0.04)  P=0.55 |
| History of IDU (no vs. yes) | 0.03 (-0.06, 0.11)  P=0.52 | 0.03 (-0.04, 0.11)  P=0.36 | 0.06 (-0.05, 0.17)  P=0.26 | 0.09 (0.00, 0.18)  P=0.05 |
| CD4:CD8 ratio | 0.01 (-0.05, 0.07)  P=0.67 | 0.03 (-0.03, 0.08)  P=0.33 | -0.03 (-0.11, 0.05)  P=0.42 | 0.04 (-0.03, 0.10)  P=0.27 |
| Nadir CD4+ T-cell count (per 100 cells/μl) | 0.01 (-0.00, 0.03)  P=0.12 | 0.00 (-0.01, 0.02)  P=0.66 | 0.01 (-0.01, 0.03)  P=0.4 | 0.01 (-0.01, 0.02)  P=0.34 |
| Years since HIV diagnosis (per 5-year) | 0.02 (0.01, 0.04)  P=0.01 | 0.02 (0.01, 0.04)  P<0.001 | 0.04 (0.01, 0.06)  P<0.001 | 0.03 (0.01, 0.05)  P<0.001 |
| Prior AIDS event (no vs. yes) | -0.02 (-0.07, 0.03)  P=0.49 | 0.01 (-0.04, 0.05)  P=0.80 | 0.01 (-0.06, 0.08)  P=0.73 | -0.02 (-0.08, 0.04)  P=0.56 |
